# Sex Differences in Resting-State Functional Connectivity of the Cerebellum in Autism Spectrum Disorder

**DOI:** 10.3389/fnhum.2019.00104

**Published:** 2019-04-05

**Authors:** Rachel E. W. Smith, Jason A. Avery, Gregory L. Wallace, Lauren Kenworthy, Stephen J. Gotts, Alex Martin

**Affiliations:** ^1^Laboratory of Brain and Cognition, National Institute of Mental Health (NIMH), National Institutes of Health, Bethesda, MD, United States; ^2^Department of Speech, Language, and Hearing Sciences, The George Washington University, Washington, DC, United States; ^3^Children’s National Health System, Washington, DC, United States

**Keywords:** autism, fMRI, cerebellum, sex differences, connectivity, resting state

## Abstract

Autism spectrum disorder (ASD) is more prevalent in males than females, but the underlying neurobiology of this sex bias remains unclear. Given its involvement in ASD, its role in sensorimotor, cognitive, and socio-affective processes, and its developmental sensitivity to sex hormones, the cerebellum is a candidate for understanding this sex difference. The current study used resting-state functional magnetic resonance imaging (fMRI) to investigate sex-dependent differences in cortico-cerebellar organization in ASD. We collected resting-state fMRI scans from 47 females (23 ASD, 24 controls) and 120 males (56 ASD, 65 controls). Using a measure of global functional connectivity (FC), we ran a linear mixed effects analysis to determine whether there was a sex-by-diagnosis interaction in resting-state FC. Subsequent seed-based analyses from the resulting clusters were run to clarify the global connectivity effects. Two clusters in the bilateral cerebellum exhibited a diagnosis-by-sex interaction in global connectivity. These cerebellar clusters further showed a pattern of interaction with regions in the cortex, including bilateral fusiform, middle occipital, middle frontal, and precentral gyri, cingulate cortex, and precuneus. *Post hoc* tests revealed a pattern of cortico-cerebellar *hyperconnectivity* in ASD females and a pattern of *hypoconnectivity* in ASD males. Furthermore, cortico-cerebellar FC in females more closely resembled that of control males than that of control females. These results shed light on the sex-specific pathophysiology of ASD and are indicative of potentially divergent neurodevelopmental trajectories for each sex. This sex-dependent, aberrant cerebellar connectivity in ASD might also underlie some of the motor and/or socio-affective difficulties experienced by members of this population, but the symptomatic correlate(s) of these brain findings remain unknown.

Clinical Trial Registration: www.ClinicalTrials.gov, NIH Clinical Study Protocol 10-M-0027 (ZIA MH002920-09) identifier #NCT01031407

## Introduction

Autism spectrum disorder (ASD) is a heterogeneous, neurodevelopmental condition characterized by social-communicative impairments and restricted/repetitive or stereotyped behaviors (American Psychiatric Association, 2013). Diagnostic symptomatology can be reliably detected as early as 24 months (Daniels and Mandell, 2014), which suggests that etiological factors may be present very early in development. Epidemiologically, ASD also has a strong male preponderance, with only one female diagnosed for every three to four males (Lai et al., 2015a; Centers for Disease Control and Prevention, 2017). This uneven sex ratio has resulted in decades of ASD research that focuses almost exclusively on males, thereby limiting the generalizability of published findings, and leaving females with this disorder underrepresented and poorly understood. However, recent years have witnessed an uptick in the number of clinical and preclinical studies probing the male bias in ASD’s prevalence, and speculating about how it might be related to different neurobiological factors (Lai et al., 2015a, 2017), as well as numerous cultural and diagnostic biases (Dworzynski et al., 2012; Lai et al., 2015b).

Researchers have developed two theoretical biological hypotheses to explain the male prevalence in ASD diagnoses (Lai et al., 2015a, 2017). First, certain neurodevelopmental mechanisms may put males at a heightened risk of being diagnosed with ASD. Second, other mechanisms may play a protective role for females, thereby putting them at a comparatively lower risk (Robinson et al., 2013, or for review see Lai et al., 2015a, 2017; McCarthy and Wright, 2017).

A substantial amount of research has accumulated, illustrating the heritability of ASD, highlighting a large number of candidate genes, and unraveling aberrant patterns of neural organization associated with this disorder, but mechanisms responsible for ASD’s male preponderance remain poorly understood (Taniai et al., 2008; McCarthy and Wright, 2017). Some studies have found ASD’s heritability to be stronger in females than in males, and that females appear to require a larger number of genetic mutations or related polymorphisms to be present before they reach the threshold for an ASD phenotype (Robinson et al., 2013; Jacquemont et al., 2014). This increased genetic burden could be interpreted as evidence for a female protective effect in action (Lai et al., 2017; McCarthy and Wright, 2017). However, males’ vulnerability for developing ASD cannot be explained by any individual genetic risk factors or neurogenetic conditions such as fragile X syndrome, and there is a lack of a conclusive sex bias in any of the implicated candidate genes (Iossifov et al., 2014; Hampson and Blatt, 2015; Sanders et al., 2015). If individual genes and rare neurogenetic syndromes cannot successfully explain the heightened risk for ASD in males, what other neurodevelopmental mechanisms might help to account for the disorder’s male-biased prevalence?

While the answer to this question remains equivocal, some researchers are turning their empirical attention toward the cerebellum for several reasons (Dean and McCarthy, 2008; Hampson and Blatt, 2015; Lai et al., 2017). First, abnormalities in cerebellar histology, structure, and function have all been associated with ASD, as well as with other neurodevelopmental disorders whose prevalence or age of onset are determined by biological sex (e.g., attention-deficit hyperactivity disorder, or ADHD, schizophrenia, and dyslexia; Courchesne et al., 1988; Nguon et al., 2005; McCarthy and Wright, 2017). In fact, cerebellar pathology carries the highest risk ratio for ASD of any single, non-heritable factor (Wang et al., 2014). Postmortem and magnetic resonance imaging (MRI) studies of ASD have noted a reduction in Purkinje and granular cells, volumetric changes, and abnormal task-based activation patterns in this region (Nguon et al., 2005; Bloss and Courchesne, 2007; Hampson and Blatt, 2015). In addition, congenital or acquired cerebellar injury often results in cognitive and affective deficits similar to those seen in ASD (Schmahmann, 2010; Hampson and Blatt, 2015).

Another motivating factor for investigating the cerebellum in the context of sex differences in ASD is the simultaneous co-expression of ASD candidate genes and rising gonadal steroids that promote neural growth (Koibuchi and Ikeda, 2013) in this same region during development (Menache et al., 2013; Willsey et al., 2013; Wang et al., 2014). Studies conducted using selectively bred mice have suggested that sex steroids mediate cerebellar neurodegeneration, and also that estrogens may have a protective effect on localized neural development (Dean and McCarthy, 2008; Biamonte et al., 2009). Human studies have also established a link between androgen levels *in utero* and ASD diagnoses, and have also noted endocrine abnormalities in people with this disorder (Ingudomnukul et al., 2007; Bejerot et al., 2012; Baron-Cohen et al., 2015; McCarthy and Wright, 2017). Therefore, the coincidence of gene expression and sex-dependent fluctuations in gonadal steroids could be one mechanism by which early steroidogenic dysregulation can lead to ASD symptomatology.

Recent mapping of the cerebellum’s functional organization has provided a rough framework for how these early chemical deviations can precipitate later widespread changes in cortical organization seen in this disorder (see Picci et al., 2016; Mash et al., 2018) for recent reviews of neuroimaging findings in ASD). Indeed, resting-state functional MRI (fMRI) has allowed researchers to spatially map functional connections across the cerebellum by measuring their baseline coherence with other parts of the cortex (Buckner et al., 2011). This has demonstrated that different subdivisions of the cerebellum are functionally connected to a range of cortical areas whose functions include: socio-affective processing (Buckner et al., 2011; Buckner, 2013; Baumann et al., 2015), executive function, theory of mind, language, auditory discrimination (Baumann and Mattingley, 2010), visuospatial processing including biological motion (Baumann et al., 2015), and of course, somatomotor functions (Buckner et al., 2011; Wang et al., 2014). Individuals with ASD experience deficits in many, or all, of these behavioral domains, and sex-dependent alterations in cerebellar-cortical network dynamics could be partially responsible.

Researchers have used functional and microstructural data to explain the relationship between cerebellar development and the extensive cortical changes associated with ASD (Wang et al., 2014; D’Mello and Stoodley, 2015). A popular theory is that injury to the cerebellum, either congenital or acquired *via* sex-dependent hormonal or environmental factors, interrupts closed-loop cerebellar-cortical circuits while the brain’s functional architecture is still fragile. With compromised structural integrity, the cerebellum acts “upstream” (Wang et al., 2014) to reshape the brain’s functional organization, and this ultimately leads to the “downstream” cortical and behavioral abnormalities seen in ASD (Olivito et al., 2018). Although studies are inconsistent with respect to directionality, there do appear to be sustained changes in cerebellar-cortical functional connectivity (FC) in ASD (Noonan et al., 2009; Khan et al., 2015; Olivito et al., 2018), but whether these changes manifest differently in ASD males and ASD females remains unknown.

There is limited functional neuroimaging data on females with ASD, and studies exploring how sex mediates resting state FC between the cerebellum and the cortex are even rarer. Many fMRI studies of ASD have reported findings in the cerebellum (Hull et al., 2017), but these results are inconsistent, often overshadowed by robust cortical differences, and exclusive representative of ASD males. In one study with a predominantly male sample, the cerebellum exhibited a pattern of reduced FC in ASD compared to controls, much like the connectivity reductions seen in social brain areas (Gotts et al., 2012). However, two other male-biased studies found the opposite pattern of increased cerebro-cerebellar FC, as well as atypical lateralization and “non-canonical” organization (Noonan et al., 2009; Khan et al., 2015).

One recent resting state study reported increased FC between the cerebellum and a seed in the left superior temporal sulcus in females with ASD, compared to controls, but there was no subsequent discussion of this finding (Alaerts et al., 2016). The same study also reported diagnosis-by-sex interactions between the left cerebellar vermis VIII, and the right precuneus and left inferior frontal gyrus, whereby in ASD, females displayed increased FC and males showed decreased FC. While this specific finding was also not discussed, the authors concluded that FC profiles in females with ASD were consistent with “neural masculinization,” or a shift towards the organizational patterns seen in healthy males, whereas males with ASD displayed a “neural feminization” or FC more similar to healthy females, also referred to as “gender incoherence” (Alaerts et al., 2016; see also Baron-Cohen, 2002; Bejerot et al., 2012; Floris et al., 2018). These findings were inconsistent with the extant “extreme male brain” theory of ASD (Baron-Cohen, 2002).

The goal of the current study was to examine differences in cerebellar-cortical organization in males and females with ASD, by identifying areas that exhibit a diagnosis-by-sex interaction in their patterns of resting-state FC. However, given the paucity of information about the similarity of ASD male and female patterns of altered brain dynamics, we employed a data-driven, whole-brain approach to the analyses, permitting us to identify results outside of the cerebellum, if they were to exist.

## Materials and Methods

### Participants

Participants included 88 (24 female, 65 male) typically developing (TD) Individuals (mean age 21.51 ± 7.54; age range 10–54), and 79 (23 female, 56 male) individuals diagnosed with ASD (mean age 21.63 ± 10.79; age range 11–62; see Table 1 for a summary of participant demographic information). While these age ranges appear extremely broad, they were matched across all experimental groups, and most participants in all groups ranged between 10 and 25 years of age (see Supplementary Figure S1 for age histograms by group). Exclusion criteria included the presence of any neurogenetic disorders such as fragile X syndrome, congenital or traumatic brain injuries, full-scale intelligence quotient (FSIQ) scores below 80, and psychiatric disorders in the TD group. All ASD participants met DSM-5 diagnostic criteria for ASD as assessed by an experienced clinician. Additionally, all participants with ASD met diagnostic criteria for “broader ASD” according to guidelines established by the National Institute of Child Health and Human Development/National Institute on Deafness and Other Communication Disorders Collaborative Programs for Excellence in Autism (Lainhart et al., 2006), and based on scores from either the Autism Diagnostic Interview (ADI or ADI-R; Le Couteur et al., 1989; Lord et al., 1994), or the Autism Diagnostic Observation Schedule (ADOS or ADOS-2; Lord et al., 2000) administered by research-reliable clinicians.

**Table 1:** Participant demographic characteristics.

	ASD females (*n* = 23)	ASD males (*n* = 56)	TD females (*n* = 24)	TD males (*n* = 65)	*P* value
Age (years)	21.84 (13.94)	21.63 (9.27)	21.80 (10.30)	21.41 (6.33)	*p* = 0.1586^i^
Head motion^a^	0.072 (0.059)	0.073 (0.063)	0.067 (0.034)	0.069 (0.035)	*p* = 0.9465^i^
FSIQ	114.38 (16.43)	109.62 (16.13)	113.29 (12.64)	116.32 (11.18)	*p* = 0.1324^i^
Social responsiveness scale (sum)	96.90 (26.68)^b^	88.30 (31.17)^c^			*p* = 0.3314^j^
ADI social	17.46 (4.98)^d^	21.30 (4.32)^e^			*p* < 0.0505^j^
ADI verbal communication	13.46 (4.37)^d^	16.02 (4.37)^e^			*p* < 0.0494^j^
ADI restricted/repetitive behaviors	5.00 (2.35)^d^	5.47 (2.62)^e^			*p* = 0.9337^j^
ADOS social + communication	9.56 (2.18)^f^	12.31 (3.25)^g^			*p* < 0.0106^j^
ADOS stereotyped behaviors	1.39 (1.14)^f^	1.47 (1.47)^g^			*p* = 0.6236^j^
ADOS-2 social affect	12.25 (5.50)^h^				
ADOS-2 restricted/repetitive behavior	3.5 (0.58)^h^				

*FSIQ, Full-Scale Intelligence Quotient (Standard Score). Data are mean (standard deviation). ^a^motion is measured *via* frame-wise displacement in mm/TR ^b^*n* = 20. ^c^*n* = 53. ^d^*n* = 16. ^e^*n* = 50. ^f^*n* = 18. ^g^*n* = 55. ^h^*n* = 3. ^i^Kruskal-Wallis Test. ^j^Wilcoxon Rank Sum Test*.

This study was carried out in accordance with the recommendations of the Institutional Review Board of the National Institute of Mental Health with written informed consent from all subjects and/or parents or legal guardians where necessary. All subjects gave written informed consent or assent in accordance with the Declaration of Helsinki. The protocol (NIH Clinical Study Protocol 10-M-0027, ZIA MH002920-09) was approved by the Institutional Review Board of the National Institute of Mental Health. Participants completed measures of FSIQ, using either the Wechsler Abbreviated Scale of Intelligence, the Wechsler Abbreviated Scale of Intelligence-II, the Wechsler Intelligence Scale for Children-IV, or the Wechsler Intelligence Scale for Children-V. Due to the exclusion criteria mentioned above, the current study only examined cases of ASD where IQ scores were average or above average, which helped to maintain comparability of FSIQ across the ASD and TD groups.

### Image Acquisition

Structural and fMRI data were acquired using a GE Signa 3T whole-body MRI scanner at the NIH Clinical Center NMR Research Facility. During all scans, a GE 8-channel receive-only head coil was used, with an acceleration (SENSE) factor of 2. First, T1-weighted anatomical images magnetization prepared rapid acquisition gradient echo (MPRAGE) were collected for each participant (124 axial slices, 1.2 mm slice thickness, field of view = 24 cm, 224 224 acquisition matrix), followed by resting-state scans, which measure slow, spontaneous, blood-oxygenation-level-dependent (BOLD) fluctuations. Participants were instructed to lie quietly and fix their gaze on a central cross. Each gradient-echo echo-planar rest scan lasted 8 min and 10 s (140 consecutive volumes with whole-brain coverage, repetition time = 3,500 ms, echo time = 27 ms, 90° flip angle, 42 axial contiguous interleaved slices per volume, 3.0 mm slice thickness, 22 cm field of view, 128 × 128 acquisition matrix, 1.7 mm × 1.7 mm × 3.0 mm voxel size). Cardiac and respiratory signals were recorded during the resting-state scan for later regression during image preprocessing.

#### fMRI Preprocessing

Echo-planar images (EPIs) were preprocessed using the Analysis of Functional NeuroImages (AFNI) software package (Cox, 1996). The first three EPI volumes were removed from the scan, after which AFNI’s 3dDespike was applied in order to attenuate any extreme deviations in the voxel-wise signals. The remaining EPI volumes were then slice-time corrected (to slice-time 0) and co-registered with their respective anatomical scans (MPRAGEs). Scans were blurred using a 6 mm isotropic full-width at half-maximum Gaussian kernel and rescaled to percent signal change by normalizing to each voxel’s mean BOLD signal intensity. Finally, in preparation for statistical group comparisons, all scans were resampled to 3.0 mm^3^ voxels, and transformed into standard Talairach and Tournoux (1988) space.

AFNI’s ANATICOR protocol was used to regress nuisance artifacts from the EPI data (Jo et al., 2010, 2013; see also Gotts et al., 2012; Berman et al., 2016). This process began by segmenting the MPRAGE scans, which was done using FreeSurfer (Fischl et al., 2002). Masks were then made for ventricles and white matter, resampled to the EPI resolution, and eroded by 1 voxel to prevent partial volume effects with the gray matter. Before smoothing, a single average nuisance time series for the ventricles was calculated using the EPI data, along with a localized average of white matter, centered on each voxel and averaged within a 15 mm-radius sphere. Including a localized average white matter signal has been shown to reduce the dependence of FC on transient motion, as reflected in motion-censoring analyses (e.g., Jo et al., 2013). Using the respiration and cardiac data collected during the scan, respiration volume per time (RVT; Birn et al., 2008) and Retroicor (Glover et al., 2000) regressors were generated. Before least-squares model fitting to every voxel’s time series, nuisance variables were detrended with fourth-order polynomials. The full nuisance regression model, therefore, contained one localized white matter time series, one average ventricle time series, six motion parameters, eight Retroicor time series (four cardiac, four respiration), five RVT time series, along with a 4th-order polynomial baseline model. Residual time series were generated by subtracting the best fit time series of this nuisance model from the full, volume-registered time series.

#### fMRI Analyses

All analyses were performed using AFNI (Cox, 1996) and MATLAB. First, mean “connectedness” was calculated (Pearson’s r) at every gray matter voxel for each participant (e.g., Cole et al., 2010; Salomon et al., 2011; Gotts et al., 2012). This technique assigns each voxel a single value representing the mean of all correlations between that voxel and the rest of the voxels in the gray matter brain mask. Using Fisher-z transformed subject-level whole-brain connectedness-maps, a 2 × 2 linear mixed effects analysis (AFNI’s 3dLME) was run in order to identify any regions showing a diagnosis-by-sex interaction, by specifying the following model: (ASD males—ASD Females) × (TD males—TD females). Age, motion (AFNI’s @1dDiffMag, comparable to mean frame-wise displacement in units of mm/TR), and global correlation level (GCOR; Saad et al., 2013) were all included in the model as nuisance covariates. Small volume correction was performed for the cerebellum using AFNI’s 3dClustSim with empirical autocorrelation function (ACF) parameters using cluster size (Cox et al., 2017). The cerebellum mask used for this correction was comprised of only those voxels that contained full EPI data in at least 85 percent of the participants in all groups.

To elucidate the initial connectedness findings, surviving clusters from the first linear mixed effects analysis were then used as seed regions for subsequent analyses by creating a 6 mm radius sphere around their peak coordinates (see Gotts et al., 2012; Berman et al., 2016; Stoddard et al., 2016 for further discussion). Mean time series within the spherical masks were extracted from the cleaned residual time series for every subject. These average time series were then correlated with the rest of the voxels in a whole-brain mask. An additional 2 × 2 linear mixed effects analysis was run for each cerebellar seed on the resulting seed-based correlation maps, to determine which other cortical or subcortical regions might be driving the interaction effect found in the primary analysis of connectedness. This approach is analogous to *post hoc* testing following the detection of a significant main effect in an ANOVA, as the locations driving connectedness effects remain unclear without further seed testing. The seed tests were corrected for multiple comparisons using cluster size within a whole-brain mask (Cox et al., 2017), with the adjustment that the maps were corrected to *P* < 0.05 divided by the number of seed tests being performed (Bonferroni), thereby adjusting for whole-brain voxel-wise tests and the number of seed tests at the same time (see Gotts et al., 2012 for further discussion). The seed tests were also evaluated for any noise bias in the voxel selection using a leave-one-out cross-validation approach (see Supplementary Materials). Lastly, pair-wise correlations were calculated among all possible region-to-region pairs for three contrasts: ASD—TD within each sex, and diagnosis-by-sex. To do this, masks were created for the 13 regions that survived correction, and average time series were extracted for each. With two cells for the cerebellar seeds included, the 15 × 15 correlation matrices were thresholded below false discovery rate (FDR)-corrected levels (*P* < 0.0069 for *q* < 0.05). For the within-sex correlation matrices, *p*-values only within those region-to-region combinations that exhibited an FDR-corrected diagnosis-by-sex interaction were considered.

To investigate the extent to which the data support prevailing theories of either “gender incoherence” (Bejerot et al., 2012; Alaerts et al., 2016) or extreme masculinization (Baron-Cohen, 2002; Baron-Cohen et al., 2015) of the brain in ASD, two different measures were applied to the set of region-to-region pairs exhibiting a significant diagnosis-by-sex interaction: (1) pearson correlation of each individual ASD participant with the mean pattern of TD males and females; and (2) the Euclidean distance between each ASD participant with the mean pattern of TD males and females. The correlation measure differs mainly from the Euclidean distance measure in that the mean region-by-region FC values were removed, evaluating only the pattern similarity rather than the mean differences that helped to define the diagnosis-by-sex interactions in each region-by-region combination. Correlations and Euclidean distances from TD males and females were compared by paired *t*-tests after checking for normality.

## Results

Matching across the experimental groups on nuisance variables was evaluated with the Kruskal-Wallis Test. Results indicated that the four groups were indeed adequately matched on age, head motion, and IQ (*p* = 0.1586, *p* = 0.9465, and *p* = 0.1324, respectively; see Table 1). When comparing ADOS and ADI subscale scores between males and females with Wilcoxon Rank Sum tests (also in Table 1), some modest significant differences emerged, most notably in the Social + Communication subscale of the ADOS (*p* < 0.0106). In this case, ASD males appeared to have more severe deficits in this category than ASD females, although both groups exhibited impairment. However, these differences were not observed in the scores on the Social Responsiveness Scale (SRS), which is designed to be a more cardinal measure for use in correlational and other behavioral analyses.

**Figure 1:**
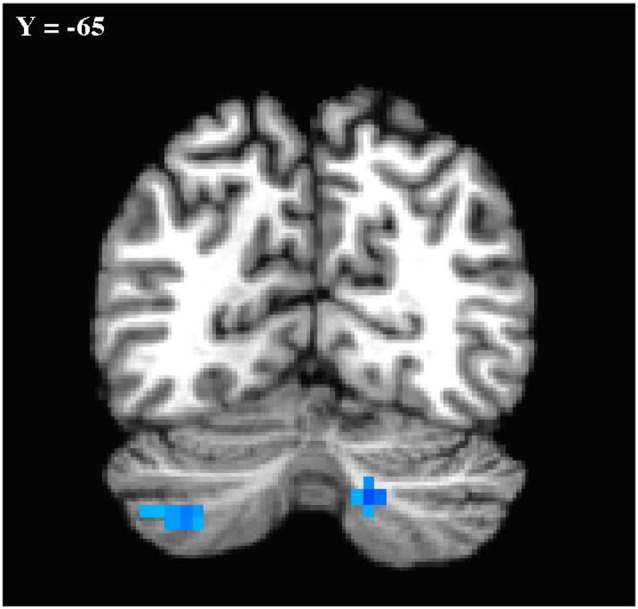
Cerebellar clusters exhibiting a diagnosis-by-sex interaction. Shown in standard Talairach Tournoux space. Left is shown on the left. Mode contrast = autism spectrum disorder (ASD males—ASD Females) × typically developing (TD males—TD females). Clusters shown were significant at *p* < 0.05 with small volume correction for the cerebellum.

**Figure 2:**
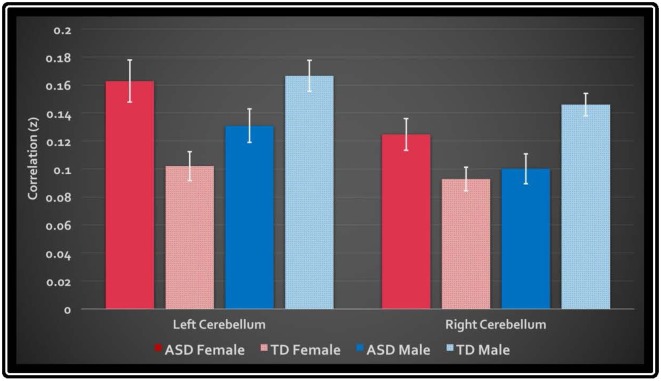
Mean global connectedness values for left and right cerebellar clusters. The *y*-axis displays the average z-transformed correlation coefficient of the cluster’s mask with every other voxel in the brain. White bars denote standard error.

In the primary analysis of global connectedness, significant diagnosis-by-sex effects were initially found in four clusters (voxel-wise threshold of *p* < 0.005, uncorrected): two in the bilateral cerebellum (left crus II and right lobule VIIIA/B, just lateral to the vermis), and two in the bilateral superior temporal gyri. While none of these clusters survived whole-brain cluster-size correction, after small volume correction for the cerebellum, both left and right cerebellar clusters survived (corrected to *p* < 0.05; Figure 1). To establish the direction of the interaction, average time courses were extracted for each cerebellar cluster. Both clusters exhibited a similar crossover interaction pattern of mean connectedness with the rest of the cortex, whereby males with ASD displayed significantly *reduced* FC compared to their TD male counterparts, and females with ASD displayed significantly *increased* connectivity compared to TD females (Figure 2). Subsequent seed-based analyses were only performed on the two significant cerebellar clusters.

**Figure 3:**
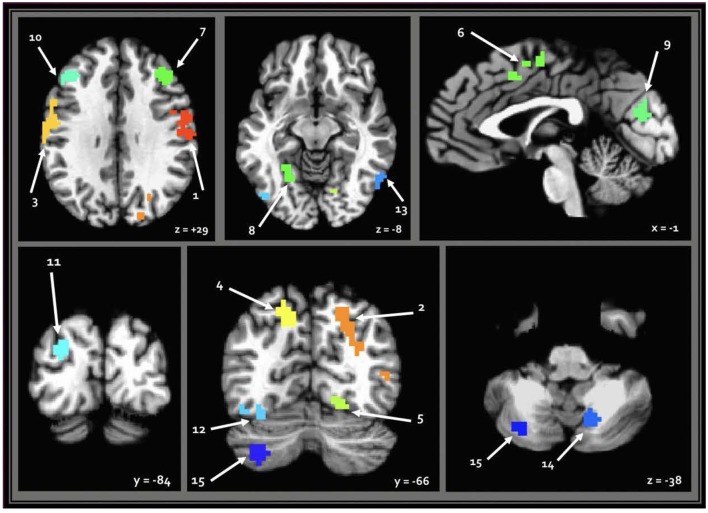
Regions resulting from seed-based analysis showing diagnosis-by-sex interaction. Cerebellar seeds are pictured in the lower right panel. Left is shown on the left, and results are in standard Talairach-Tournoux space. Refer to Table 2 for key of numeric region labels.

When seeding from the right (VIII) cerebellar cluster, 12 predominantly bilateral regions emerged, exhibiting a corresponding diagnosis-by-sex interaction. These were located in the bilateral precentral, middle occipital, middle frontal, fusiform, and cingulate gyri, as well as the left cuneus, precuneus, and inferior temporal gyrus (Table 2, Figure 3). When seeding from the left (crus II) cerebellar cluster, an additional region emerged in the right precuneus. These seed-based tests only assessed relationships between the seeds in the cerebellum and the cortex.

**Table 2:** Numbered list of resulting regions from each cerebellar seed.

Region #	Region label	Peak coordinates^a^
1	R. precentral gyrus	(41, −13, 44)
2	R. precuneus	(17, −79, 35)
3	L. precentral gyrus	(−37, −10, 47)
4	L. precuneus	(−28, −46, 59)
5	R. fusiform gyrus	(23, −58, −19)
6	R/L. cingulate gyrus	(2, 11, 44)
7	R. middle frontal gyrus	(38, 32, 29)
8	L. fusiform gyrus 1	(−25, −49, −7)
9	L. cuneus	(−4, −76, 17)
10	L. middle frontal gyrus	(−37, 32, 32)
11	L. middle occipital gyrus	(−22, −85, 20)
12	L. fusiform gyrus 2	(−28, −67, −16)
13	R. middle occipital gyrus	(53, −61, 2)
14	R. cerebellum VIII	(14, −61, −37)
15	L. cerebellum crus II	(−31, −67, −40)

**Figure 4:**
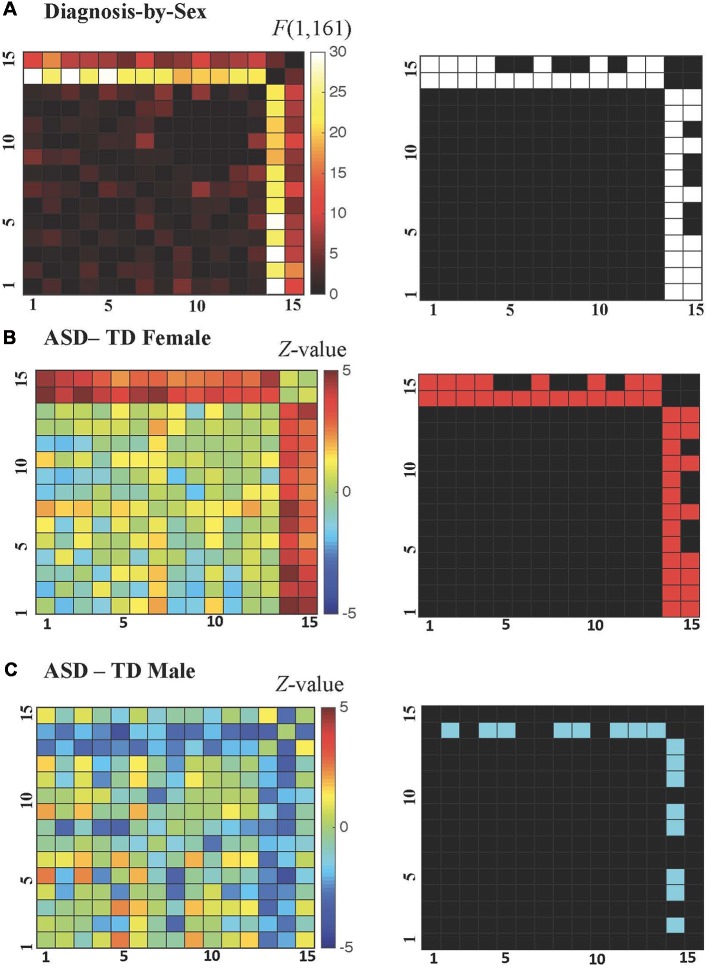
**(A–C)** Cerebellar-cortical region correlation matrices. **(A)** *F*-values for diagnosis-by-sex interaction (left), and surviving pairs at *P* < 0.0069 after false discovery rate (FDR) correction to *q* < 0.05 shown in white (right). **(B)** Z-values for ASD—TD contrast in females (left), and surviving pairs at *P* < 0.0216 (FDR-corrected to *q* < 0.05 within those combinations already FDR-corrected in the diagnosis-by-sex contrast) shown in red (right). **(C)** Z-values for ASD—TD contrast in males (left), and surviving pairs at *P* < 0.0216 (FDR-corrected to *q* < 0.05 within those combinations already FDR-corrected in the diagnosis-by-sex contrast) shown in blue (right).

*Numbered key of cortical regions resulting from seed-based tests. Numbers in parenthesis refer directly to those used in Figures 3, 4. ^a^Coordinates are *(x, y, z)*, in standard Talairach-Tournoux space, *left-posterior-inferior* orientation, peaks from cluster-size corrected seed-based maps*.

All possible regional inter-relationships were then assessed in the full region-to-region correlation matrices. However, only the cerebellum-to-cortex relationships identified in the seed-based tests survived FDR correction (*q* < 0.05), and these detected diagnosis-by-sex interactions were qualitatively similar to those identified in the earlier analyses of whole-brain connectedness (see Figure 4A), with correlation decreases observed in ASD males compared to TD males and correlation increases observed in ASD females compared to TD females. For the ASD-TD contrasts in females, all region-to-region pairs showing a significant diagnosis-by-sex interaction also showed significant increases in ASD females (Figure 4B). In contrast, for the ASD-TD contrasts in males, decreases were observed in ASD males between the right cerebellar VIII seed and the bilateral precuneus, bilateral fusiform, bilateral middle occipital gyri, left cuneus, and left inferior temporal gyrus (Figure 4C).

Given the modest differences in the subscales of the ADI and ADOS shown in Table 1, we examined whether there might be a correspondence between the ASD male/female subscale scores and the diagnosis-by-sex interactions observed in FC. No significant correlations were observed with the ADOS social+communication score and region-to-region pairs exhibiting interactions, even at a *p* < 0.05 (uncorrected) level, nor were there any differences in the slope of these relationships by group (ASD male/female). Corresponding analyses using SRS sum score also failed to yield significant results.

Finally, FC of the region-to-region pairs exhibiting a diagnosis-by-sex interaction was examined to evaluate the gender incoherence and extreme male brain theories. The gender incoherence theory predicts that ASD males should be more similar to TD females and ASD females should be more similar to TD males, whereas the extreme male brain theory predicts that both ASD males and females should be more similar to TD males—and that ASD males are further away from TD females on the sex continuum than TD males are (i.e., they are extreme male). Results for the Pearson correlation measure are shown in Table 3. On average, the pattern of cerebellar-cortical FC in ASD females was more similar to TD males rather than to TD females [paired *t*-test(22): 2.3457, *p* < 0.0284], and this result remained significant when using Euclidean distance to compare groups [paired *t*-test(22): −4.1914, *p* < 6.4891e-05; see Table 4]. ASD males had FC patterns more similar to TD males than to TD females [paired *t*-test(55): 3.3597, *p* < 0.0014], however, this effect was no longer significant or apparent when using Euclidean distance as a means of comparison. It is also worth noting that ASD females were numerically closer to TD males than ASD males were to their same-sex controls, though this failed to reach significance (*p* < 0.1974). Additionally, the numeric distance between TD males and TD females was greater than the distances between ASD males and TD females [paired *t*-test(143): −2.4207, *p* < 0.0167], and ASD males and TD males [paired *t*-test(143): −2.3234, *p* < 0.0216], indicating that ASD males lie *between* TD males and TD females with respect to pattern and magnitudes of FC.

**Table 3:** Mean correlations and tests of male/female similarity.

Clinical group	TD female	TD male	Paired *t*-test
ASD female	0.3321 (0.2354)	0.3675 (0.2065)	*t*_(22)_ = 2.3457 *P* < 0.0284*
ASD male	0.2552 (0.2010)	0.2981 (0.1722)	*t*_(55)_ = 3.3597 *P* < 0.0014*

Correlations are mean (SD) and paired *t*-tests are *t*-statistic (degrees of freedom). *Statistically significant.

**Table 4:** Euclidean distances and tests of male/female similarity.

Group	TD female	TD male	Paired *t*-test
ASD female	1.5231 (0.5853)	1.0847 (0.3110)	*t*_(22)_ = −4.1914 *p* < 6.4891e-05*
ASD male	1.2032 (0.5151)	1.2202 (0.4575)	*t*_(55)_ = 0.2253 *p* < 0.8225
TD female	1.0214 (0.3479)	1.3988 (0.5160)	
TD male	1.4515 (0.6276)	1.1228 (0.4293)	

Distances are mean (SD) and paired *t*-tests are *t*-statistic (degrees of freedom). *Statistically significant.

## Discussion

The current study sought to build upon existing literature that has explored the neurophysiology of the sex bias associated with ASD, and to focus, in particular, on the cerebellum. Early, localized co-expression of ASD candidate genes (Wang et al., 2014), regional sensitivity to the action of sex steroids (Dean and McCarthy, 2008), well-mapped connections with the rest of the cortex (Buckner et al., 2011), and consistent involvement in sex-dependent neurodevelopmental disorders (Hampson and Blatt, 2015) are all motivating factors for further investigation of cerebellum in this context. Although the current study employed a data-driven approach over the entire brain to investigate sex differences in ASD, the findings indeed highlight the role of the cerebellum.

By probing sex differences in resting state FC, the current study found that cortical-cerebellar connectivity profiles looked strikingly different in males and females with ASD. In fact, there was a significant diagnosis-by-sex interaction in mean connectedness between two sub-regions of the cerebellum and the rest of the brain. These regions (in right lobule VIII and left crus II) exhibited *hyper*-connectivity with the cortex in ASD females, and an opposing pattern of *hypo*-connectivity in ASD males. These current findings are in line with similarly opposing FC patterns that were recently reported by Alaerts et al. (2016). No cerebellar regions showed significant differences in FC that were directionally similar across sexes in the clinical groups. Elucidation of the initial interaction implicated a range of functionally diverse cortical areas. In follow-up correlation analyses, both ASD males and ASD females in the current study exhibited levels of region-to-region connectivity that more closely resembled that of TD males than TD females. However, when comparing Euclidean distances between experimental groups, the FC pattern and magnitudes of ASD males actually appeared to fall somewhere between that of TD males and TD females, rather than at the extreme end of the TD males. Therefore, with respect to ASD-related changes in cerebellar-cortical FC, the current results align more with “gender incoherence” than with an “extreme male brain” (Baron-Cohen, 2002).

Multiple theories have been proposed to account for ASD’s male preponderance. Researchers believe that male-specific risk factors, and/or female-specific protective mechanisms help to drive the imbalanced diagnostic rate (Lai et al., 2017). With those predispositions in mind, it has been proposed that females who meet the diagnostic threshold for ASD would present with more severe neurophysiological impairments than males (Lai et al., 2015a, 2017). However, results from the current study, in conjunction with other recent publications, suggest that ASD’s pathophysiology is qualitatively, rather than simply quantitatively, different in females relative to males (Alaerts et al., 2016; Lai et al., 2017).

Because alterations in androgens and estrogens have been noted in both early developmental and later phenotypic stages of ASD (Lai et al., 2017), researchers have interpreted organizational brain changes as “neural masculinization” or “feminization” (Bejerot et al., 2012; Lai et al., 2015a, 2017; Alaerts et al., 2016) or as a shift toward an “extreme male brain” (Baron-Cohen, 2002). The current study does not find clear evidence for an “extreme male brain” (Baron-Cohen, 2002) in ASD, but it does provide support for “masculinization” in the cerebellar-cortical connectivity patterns of *females* with ASD. However, in males, the picture is less straightforward, which may be a byproduct of the relatively small sample of females to which they were compared. ASD males in the current study had FC *patterns* (excluding the mean FC values) that were more similar to those of TD males than TD females, but when taking magnitude into account (Euclidean distances), this was no longer true. In addition, ASD females were actually numerically closer to TD males than ASD males are, though this failed to reach significance. Furthermore, the distance between ASD males and TD females is less than the distance between TD males and TD females, again emphasizing that they actually lie somewhere between TD males and females in terms of FC magnitudes and patterns rather than at the extreme male end of the continuum. This finding is inconsistent with the “extreme male brain” theory, and while it does not provide robust, unequivocal evidence for the “gender incoherence” reported in other studies, it is more in line with “gender incoherence” theory than with the former theoretical framework for ASD.

Other studies have offered support for the “gender incoherence” theory (Bejerot et al., 2012; Alaerts et al., 2016) rather than the “extreme male brain” (Baron-Cohen, 2002). In addition to patterns of neural activity, Bejerot et al. (2012) also saw a pattern of male/female “gender incoherence” in the physical appearance and endocrine profiles of participants with ASD, noting in a study that females had less feminine facial features, and higher levels of testosterone than controls, while males had less masculine voices and body characteristics, but no change in testosterone levels. In contrast, another recent study reported exaggerated masculine features in both males and females with the disorder (Tan et al., 2017), so the link between gendered brain organization, hormonal dysregulation, and external features is not straightforward. It is likely that multiple converging mechanisms are responsible for the sex-based divergence in brain organization and physical features in ASD.

In the current study, diagnosis-dependent sex differences in cerebellar organization were found in two clusters, in the right lobule VIII and the left crus II. Studies have previously linked volumetric changes in these regions with ASD, consistently reporting gray matter reductions in lobule VIII (D’Mello and Stoodley, 2015), and also correlating diagnosed children’s communication difficulties with decreased gray matter in bilateral crus II and VIII (Riva et al., 2013).

The seed-based tests from each cerebellar cluster in the current study yielded a total of 13 predominantly bilateral regions underlying the interaction effects. Functional organization in the cerebellum and cortex tends to follow a predictably contralateral pattern, so the current finding of bilateral cortical regions for a unilateral (right) cerebellar seed is suggestive of broadly altered cortical-cerebellar circuitry in ASD, which is consistent with existing literature (Noonan et al., 2009; Khan et al., 2015). Cerebellar crus II is reliably connected to cortical areas involving both sensorimotor and social abilities, and previous studies have found aberrant connectivity among crus II and these regions in ASD. However, the current study only found diagnosis-dependent sex differences in FC between this cerebellar sub-region and the right precuneus. When seeding from the right lobule VIII, the remaining regions were in cortical areas associated with somatomotor, visuospatial, perceptual, and executive functions. Lobule VIII-A/B, also overlaps with areas previously shown to be involved in auditory processing and discrimination (Baumann and Mattingley, 2010), which is also often impaired in ASD.

It is doubtful that the results found in the current study provide a full picture of the sex-based cortical-cerebellar abnormalities in ASD, and based on the disorder’s symptomatic heterogeneity, there may be individual differences in the specific subdivisions of the cerebellum that are most affected. It is important to note that the current sample was the only representative of ASD phenotypes *without* intellectual disability, and this limits the generalizability of the current findings to only a subset of the broader ASD population. Cerebellar structure and function have both been linked with IQ (Hogan et al., 2011), and importantly, a recent study also noted significant sex differences in the degree to which cerebellar-cortical connectivity corresponded with IQ in the general population (Pezoulas et al., 2017). These researchers found that only females showed a significant correlation between cerebellar network efficiency and IQ. Neuroimaging studies that incorporate more cognitively diverse ASD populations would help to shed light on whether sex differences in cerebellar architecture look different in groups with more severe cognitive deficits.

There is extensive evidence to suggest that the cerebellum, through its functional relationship with the cortex, plays an important role in a range of behaviors, from auditory processing, to perceiving biological motion, to labeling emotional faces (Baumann and Mattingley, 2010; Buckner, 2013; Baumann et al., 2015; Jack et al., 2017). The right cerebellar cluster, in VIII-A/B (Buckner et al., 2011) partially overlaps with areas previously shown to be involved in auditory processing (Baumann and Mattingley, 2010). This has interesting implications, given that over- or under-sensitivity to auditory stimuli is a very common ASD symptom. Unfortunately, without behavioral measures or an experimental task to probe this in the current study, it remains unclear whether there is a direct relationship between the degree of cerebellar-cortical connectivity and the ASD sensory phenotype.

Because there is limited clinical literature on sex differences in ASD phenotypes, it is difficult to speculate about potential behavioral correlates for the current study’s brain-based results. In a recent qualitative analysis, clinicians reported that sex differences were more exaggerated in certain domains of ASD symptomatology (Halladay et al., 2015; Jamison et al., 2017). In particular, there were larger behavioral discrepancies in repetitive, restricted behaviors, and fewer in social communication, with males tending to exhibit more severe motor symptoms (Halladay et al., 2015; Jamison et al., 2017). Another recent study found that in parent reports of executive function, females with ASD showed greater difficulties than males (White et al., 2017). Naturally, it is important to consider the role that social expectations play in assessing males and females with ASD, and it is also important to remember that ASD diagnostic measures were not developed or normed with female samples, which is yet another limitation in the accurate identification of sex differences in this disorder.

Given the multitude of higher functions in which the cerebellum is involved, including social and motor processes, it would be useful to probe sex-based behavioral differences further and attempt to relate them to resting state and task-based activity in different sub-regions of the cerebellum. This will eventually provide a more complete picture of the cortical-cerebellar changes associated with ASD, and will also delineate those changes that differ significantly between males and females.

## Limitations

The first major limitation of the current study was the relatively small sample of females who were recruited and scanned. Given the disproportionate number of males and females diagnosed with ASD, the uneven sex ratio seen in the current sample is not surprising, and the smaller number of females may help to explain the lack of other significant cortical findings in the initial diagnosis-by-sex interaction analysis (i.e., producing an expectation of Type II rather than Type I errors).

Another limiting factor was the exclusion of ASD participants with cognitive impairments. These exclusion criteria allowed for the comparison of clinical and healthy control groups without the confound of IQ discrepancies, but it also compounded the challenge of recruiting a sufficient number of eligible female ASD participants. The participants included in the current study, therefore, represent only a subgroup of the broader ASD population, and the current results should not be extrapolated to the full, heterogeneous spectrum of ASD. This is particularly relevant in light of the current study’s cerebellar findings, as IQ has been linked to cerebellar structure, function, and connectivity profiles (Hogan et al., 2011; Pezoulas et al., 2017). In future studies, it would be helpful to investigate whether the same crossover interaction pattern observed in the current study persists in a more heterogeneous ASD sample, or in a set of low-IQ samples.

In addition to exploring cerebellar-cortical organization in ASD within a subgroup that *does* have intellectual disability, it would also be informative to gather similar data during early childhood, or across multiple time points in a longitudinal design. The current sample consisted predominantly of adults and included no young children, so this study’s findings only provide a static snapshot of cerebellar-cortical organization in adults with this disorder. Without developmental context, it cannot be determined wither these sex-dependent changes are stable in ASD, or whether they diverge in males and females earlier in development. Furthermore, given the potential influence of sex steroids on early cerebellar development, it would also be worthwhile to relate the functional architecture of the cerebellum to reproductive hormone levels and to establish whether there is a direct relationship between the two at any point in development.

An important limitation of the current study is that it does not provide insight into the symptomatic correlates of the observed sex differences cerebellar-cortical connectivity. It would be difficult to select a single behavioral domain for investigation as a correlate of the brain-based findings, due to the functional diversity of cortical regions implicated, and the wide range of behaviors that the cerebellum subserves. Additionally, the measures by which both males and females are assessed have been designed and normed using male samples, which calls into question their validity and sensitivity to a specifically female ASD phenotype.

## Conclusion

To summarize, the current findings are in agreement with recent studies that have found functional *hyper*-connectivity in the brains of ASD females, and *hypo*-connectivity in ASD males (Alaerts et al., 2016), and when focusing specifically on cerebellar-cortical FC, are more consistent with the “gender incoherence theory” (Bejerot et al., 2012; Alaerts et al., 2016) than with the “extreme male brain” (Baron-Cohen, 2002). The current results also provide support for the ongoing investigation of the cerebellum as a hub for both early and sustained sex differences in ASD, and finally, they raise questions about the possible behavioral correlates of sex-dependent abnormalities in cortical-cerebellar organization that warrant further exploration.

## Author Contributions

RS initiated the project, preprocessed the data, ran a majority of the analyses, interpreted the results, and wrote the manuscript draft. AM and SG assisted in conceptualizing the study’s design and interpreting the results, and also made major written contributions to the manuscript. JA and SG helped to set up image preprocessing pipelines, select appropriate computational approaches for data analysis, configure scripts for multiple phases of the analyses. LK and GW were instrumental in recruiting and collecting data from participants in the clinical groups, as well as offering clinical interpretations and critical manuscript revisions.

## Conflict of Interest Statement

The authors declare that the research was conducted in the absence of any commercial or financial relationships that could be construed as a potential conflict of interest.

## Abbreviations

ACF: autocorrelation function
ADI: Autism Diagnostic Interview
ADOS: Autism Diagnostic Observation Schedule
AFNI: Analysis of Functional NeuroImages
ASDs: autism spectrum disorder(s)
BOLD: blood oxygen level dependent
FC: functional connectivity
FDR: false detection rate
fMRI: functional magnetic resonance imaging
FSIQ: Full-Scale Intelligence Quotient
IQ: Intelligence Quotient
MPRAGE: magnetization prepared rapid acquisition gradient echo
MRI: magnetic resonance imaging
RVT: respiration volume per time
TD: typically developing.

## References

[B1] Alaerts K. Swinnen S. P. Wenderoth N. (2016). Sex differences in autism: a resting-state fMRI investigation of functional brain connectivity in males and females. Soc. Cogn. Affect. Neurosci. 11, 1002–1016. 10.1093/scan/nsw027 26989195 PMC4884321

[B2] American Psychiatric Association (2013). Diagnostic and Statistical Manual of Mental Disorders. 5th Edn. Arlington, VA: American Psychiatric Publishing.

[B3] Baron-Cohen S. (2002). The extreme male brain theory of autism. Trends Cogn. Sci. 6, 248–254. 10.1016/s1364-6613(02)01904-6 12039606

[B4] Baron-Cohen S. Auyeung B. Nørgaard-Pedersen B. Hougaard D. M. Abdallah M. W. Melgaard L.. (2015). Elevated fetal steroidogenic activity in autism. Mol. Psychiatry 20, 369–376. 10.1038/mp.2014.48 24888361 PMC4184868

[B6] Baumann O. Borra R. J. Bower J. M. Cullen K. E. Habas C. Ivry R.. (2015). Consensus paper: the role of the cerebellum in perceptual processes. Cerebellum 14, 197–220. 10.1007/s12311-014-0627-7 25479821 PMC4346664

[B5] Baumann O. Mattingley J. B. (2010). Scaling of neural responses to visual and auditory motion in the human cerebellum. J. Neurosci. 30, 4489–4495. 10.1523/JNEUROSCI.5661-09.2010 20335485 PMC6634498

[B7] Bejerot S. Eriksson J. M. Bonde S. Carlström K. Humble M. B. Eriksson E. (2012). The extreme male brain revisited: gender coherence in adults with autism spectrum disorder. Br. J. Psychiatry 201, 116–123. 10.1192/bjp.bp.111.097899 22500012

[B8] Berman R. A. Gotts S. J. McAdams H. M. Greenstein D. Lalonde F. Clasen L.. (2016). Disrupted sensorimotor and social-cognitive networks underlie symptoms in childhood-onset schizophrenia. Brain 139, 276–291. 10.1093/brain/awv306 26493637 PMC4719706

[B9] Biamonte F. Assenza G. Marino R. D’Amelio M. Panteri R. Caruso D.. (2009). Interactions between neuroactive steroids and reelin haploinsufficiency in Purkinje cell survival. Neurobiol. Dis. 36, 103–105. 10.1016/j.nbd.2009.07.001 19595767

[B10] Birn R. M. Smith M. A. Jones T. B. Bandettini P. A. (2008). The respiration response function: the temporal dynamics of fMRI signal fluctuations related to changes in respiration. Neuroimage 40, 644–654. 10.1016/j.neuroimage.2007.11.059 18234517 PMC2533266

[B11] Bloss C. S. Courchesne E. (2007). MRI neuroanatomy in young girls with autism: a preliminary study. J. Am. Acad. Child Adolesc. Psychiatry 46, 515–523. 10.1097/chi.0b013e318030e28b 17420687

[B12] Buckner R. L. (2013). The cerebellum and cognitive function: 25 years of insight from anatomy and neuroimaging. Neuron 80, 803–815. 10.1016/j.neuron.2013.10.044 24183029

[B13] Buckner R. L. Krienen F. M. Castellanos A. Diaz J. C. Yeo B. T. (2011). The organization of the human cerebellum estimated by intrinsic functional connectivity. J. Neurphysiol. 106, 2322–2345. 10.1152/jn.00339.2011 21795627 PMC3214121

[B14] Centers for Disease Control and Prevention (2017). Autism spectrum disorder (ASD). Data and Statistics. CDC Available online at: https://www.cdc.gov/ncbddd/autism/data.html (Accessed 2014)

[B15] Cole M. W. Pathak S. Schneider W. (2010). Identifying the brain’s most globally connected regions. Neuroimage 49, 3132–3148. 10.1016/j.neuroimage.2009.11.001 19909818

[B16] Courchesne E. Yeung-Courchesne R. Press G. A. Hesselink J. R. Jernigan T. L. (1988). Hypoplasia of cerebellar vermal lobules VI and VII in autism. N. Engl. J. Med. 318, 1349–1354. 10.1056/nejm198805263182102 3367935

[B17] Cox R. W. (1996). AFNI: software for analysis and visualization of functional magnetic resonance neuroimages. Comput. Biomed. Res. 29, 162–173. 10.1006/cbmr.1996.0014 8812068

[B18] Cox R. W. Chen G. Glen D. R. Reynolds R. C. Taylor P. A. (2017). fMRI clustering and false-positive rates. Proc. Natl. Acad. Sci. U S A 114, E3370–E3371. 10.1073/pnas.1614961114 28420798 PMC5410825

[B19] D’Mello A. M. Stoodley C. J. (2015). Cerebro-cerebellar circuits in autism spectrum disorder. Front. Neurosci. 9:408. 10.3389/fnins.2015.00408 26594140 PMC4633503

[B20] Daniels A. M. Mandell D. S. (2014). Explaining differences in age at autism spectrum disorder diagnosis: a critical review. Autism 18, 583–597. 10.1177/1362361313480277 23787411 PMC4775077

[B21] Dean S. L. McCarthy M. M. (2008). Steroids, sex and the cerebellar cortex: implications for human disease. Cerebellum 7, 38–47. 10.1007/s12311-008-0003-6 18418672 PMC2736099

[B22] Dworzynski K. Ronald A. Bolton P. Happé F. (2012). How different are girls and boys above and below the diagnostic threshold for autism spectrum disorders? J. Am. Acad. Child Adolesc. Psychiatry 51, 788–797. 10.1016/j.jaac.2012.05.018 22840550

[B23] Fischl B. Salat D. H. Busa E. Albert M. Dieterich M. Haselgrove C.. (2002). Whole brain segmentation: automated labeling of neuroanatomical structures in the human brain. Neuron 33, 341–355. 10.1016/S0896-6273(02)00569-X 11832223

[B24] Floris D. L. Lai M.-C. Nath T. Milham M. P. Di Martino A. (2018). Network-specific sex differentiation of intrinsic brain function in males with autism. Mol. Autism 9:17. 10.1186/s13229-018-0192-x 29541439 PMC5840786

[B25] Glover G. H. Li T. Q. Ress D. (2000). Image-based method for retrospective correction of physiological motion effects in fMRI: RETROICOR. Magn. Reson. Med. 44, 162–167. 10.1002/1522-2594(200007)44:1<162::aid-mrm23>3.0.co;2-e 10893535

[B26] Gotts S. J. Simmons W. K. Milbury L. A. Wallace G. L. Cox R. W. Martin A. (2012). Fractionation of social brain circuits in autism spectrum disorders. Brain 135, 2711–2725. 10.1093/brain/aws160 22791801 PMC3437021

[B27] Halladay A. K. Bishop S. Constantino J. N. Daniels A. M. Koenig K. Palmer K.. (2015). Sex and gender differences in autism spectrum disorder: summarizing evidence gaps and identifying emerging areas of priority. Mol. Autism 6:36. 10.1186/s13229-015-0019-y 26075049 PMC4465158

[B28] Hampson D. R. Blatt G. J. (2015). Autism spectrum disorders and neuropathology of the cerebellum. Front. Neurosci. 9:420. 10.3389/fnins.2015.00420 26594141 PMC4635214

[B29] Hogan M. J. Staff R. T. Bunting B. P. Murray A. D. Ahearn T. S. Deary I. J.. (2011). Cerebellar brain volume accounts for variance in cognitive performance in older adults. Cortex 47, 441–450. 10.1016/j.cortex.2010.01.001 20167312

[B30] Hull J. V. Jacokes Z. J. Togerson C. M. Irimia A. Van Horn J. D. (2017). Resting state functional connectivity in autism spectrum disorders: a review. Front. Psychiatry 7:205. 10.3389/fpsyt.2016.00205 28101064 PMC5209637

[B31] Ingudomnukul E. Baron-Cohen S. Wheelwright S. Knickmeyer R. (2007). Elevated rates of testosterone-related disorders in women with autism spectrum conditions. Horm. Behav. 51, 597–604. 10.1016/j.yhbeh.2007.02.001 17462645

[B32] Iossifov I. O’Roak B. J. Sanders S. J. Ronemus M. Krumm N. Levy D.. (2014). The contribution of de novo coding mutations to autism spectrum disorder. Nature 515, 216–221. 10.1038/nature13908 25363768 PMC4313871

[B33] Jack A. Keifer C. Pelphrey K. A. (2017). Cerebellar contributions to biological motion perception in autism and typical development. Hum. Brain Mapp. 38, 1914–1932. 10.1002/hbm.23493 28150911 PMC5342927

[B34] Jacquemont S. Coe B. P. Hersch M. Duyzend M. H. Krumm N. Bergmann S.. (2014). A higher mutational burden in females supports a “female protective model” in neurodevelopmental disorders. Am. J. Hum. Genet. 94, 415–425. 10.1016/j.ajhg.2014.02.001 24581740 PMC3951938

[B35] Jamison R. Bishop S. L. Huerta M. Halladay A. K. (2017). The clinician perspective on sex differences in autism spectrum disorders. Autism 21, 772–784. 10.1177/1362361316681481 28429618

[B36] Jo H. J. Gotts S. J. Reynolds R. C. Bandettini P. A. Martin A. Cox R. W.. (2013). Effective preprocessing procedures virtually eliminate distance-dependent motion artifacts in resting state FMRI. J. Appl. Math. 2013:935154. 10.1155/2013/935154 24415902 PMC3886863

[B37] Jo H. J. Saad Z. S. Simmons W. K. Milbury L. A. Cox R. W. (2010). Mapping sources of correlation in resting state fMRI, with artifact detection and removal. Neuroimage 52, 571–582. 10.1016/j.neuroimage.2010.04.246 20420926 PMC2897154

[B38] Khan A. J. Nair A. Keown C. L. Datko M. C. Lincoln A. J. Müller R. A. (2015). Cerebro-cerebellar resting-state functional connectivity in children and adolescents with autism spectrum disorder. Biol. Psychiatry 78, 625–634. 10.1016/j.biopsych.2015.03.024 25959247 PMC5708535

[B39] Koibuchi N. Ikeda Y. (2013). “Hormones and cerebellar development,” in Handbook of the Cerebellum and Cerebellar Disorders., eds Manto M. Schmahmann J. D. Rossi F. Gruol D. L. Koibuchi N. (Dordrecht: Springer), 319–339.

[B40] Lai M. C. Baron-Cohen S. Buxbaum J. D. (2015a). Understanding Autism in the light of sex/gender. Mol. Autism 13:24. 10.1186/s13229-015-0021-4 25973161 PMC4429357

[B42] Lai M. C. Lombardo M. V. Auyeung B. Chakrabarti B. Baron-Cohen S. (2015b). Sex/gender differences and autism: setting the scene for future research. J. Am. Acad. Child Adolesc. Psychiatry 54, 11–24. 10.1016/j.jaac.2014.10.003 25524786 PMC4284309

[B41] Lai M. C. Lerch J. P. Floris D. L. Ruigork A. N. Pohl A. Lombardo M. V.. (2017). Imaging sex/gender and autism in the brain: etiological implications. Neurosci. Res. 95, 380–397. 10.1002/jnr.23948 27870420

[B43] Lainhart J. E. Bigler E. D. Bocian M. Coon H. Dinh E. Dawson G.. (2006). Head circumference and height in autism: a study by the collaborative program of excellence in autism. Am. J. Med. Genet. A 140, 2257–2274. 10.1002/ajmg.a.31465 17022081 PMC4899843

[B44] Le Couteur A. L. Rutter M. Lord C. Rios P. Robertson S. Holdgrafer M.. (1989). Autism diagnostic interview: a standardized investigator-based instrument. J. Autism Dev. Disord. 19, 363–387. 10.1007/bf02212936 2793783

[B45] Lord C. Risi S. Lambrecht L. Cook E. H. Jr. Leventhal B. L. DiLavore P. C.. (2000). The autism diagnostic observation schedule—generic: a standard measure of social and communication deficits associated with the spectrum of autism. J. Autism Dev. Disord. 30, 205–223. 10.1023/A:1005592401947 11055457

[B46] Lord C. Rutter M. Le Couteur A. (1994). Autism Diagnostic Interview-Revised: a revised version of a diagnostic interview for caregivers of individuals with possible pervasive developmental disorders. J. Autism Dev. Disord. 24, 659–685. 10.1007/bf02172145 7814313

[B47] Mash L. E. Reiter M. A. Linke A. C. Townsend J. Müller R. A. (2018). Multimodal approaches to functional connectivity in autism spectrum disorders: an integrative perspective. Dev. Neurobiol. 78, 456–473. 10.1002/dneu.22570 29266810 PMC5897150

[B48] McCarthy M. M. Wright C. L. (2017). Convergence of sex differences and the neuroimmune system in autism spectrum disorder. Biol. Psychiatry 81, 402–410. 10.1016/j.biopsych.2016.10.004 27871670 PMC5285451

[B49] Menache I. Grange P. Larsen E. C. Banerjee-Basu S. Mitra P. P. (2013). Co-expression profiling of autism genes in the mouse brain. PLoS Comput. Biol. 7:e1003128. 10.1371/journal.pcbi.1003128 23935468 PMC3723491

[B50] Nguon K. Ladd B. Baxter M. G. Sajdel-Sulkowska E. M. (2005). Sexual dimorphism in cerebellar structure, function, and response to environmental perturbations. Prog. Brain Res. 148, 341–351. 10.1016/s0079-6123(04)48027-3 15661202

[B51] Noonan S. K. Haist F. Müller R. A. (2009). Aberrant functional connectivity in autism: evidence from low-frequency BOLD signal fluctuations. Brain Res. 1262, 48–63. 10.1016/j.brainres.2008.12.076 19401185 PMC2766184

[B52] Olivito G. Lupo M. Laghi F. Clausi S. Baiocco R. Cercignani M.. (2018). Lobular patterns of cerebellar resting state connectivity in adults with autism spectrum disorder. Eur. J. Neurosci. 47, 729–735. 10.1111/ejn.13752 29057532

[B53] Pezoulas V. C. Zervakis M. Michelogiannis S. Klados M. A. (2017). Resting-state functional connectivity and network analysis of cerebellum with respect to crystallized IQ and gender. Front. Hum. Neurosci. 11:189. 10.3389/fnhum.2017.00189 28491028 PMC5405083

[B54] Picci G. Gotts S. J. Scherf K. S. (2016). A theoretical rut: revisiting and critically evaluating the generalized under/over-connectivity hypothesis of autism. Dev. Sci. 19, 524–549. 10.1111/desc.12467 27412228

[B55] Riva D. Annunziata S. Contarino V. Erbetta A. Aquino D. Bulgheroni S. (2013). Gray matter reduction in the vermis and CRUS-II is associated with social and interaction deficits in low-functioning children with Autistic Spectrum Disorders: a VBM-DARTEL study. Cerebellum 12, 676–685. 10.1007/s12311-013-0469-8 23572290

[B56] Robinson E. B. Lichtenstein P. Anckarsäter H. Happé F. Ronald A. (2013). Examining and interpreting the female protective effect against autistic behavior. Proc. Natl. Acad. Sci. U S A 110, 5258–5262. 10.1073/pnas.1211070110 23431162 PMC3612665

[B57] Saad Z. Reynolds R. C. Jo H. J. Gotts S. J. Chen G. Martin A.. (2013). Correcting brain-wide correlation differences in resting-state fMRI. Brain Connect. 3, 339–352. 10.1089/brain.2013.0156 23705677 PMC3749702

[B58] Salomon R. Bleich-Cohen M. Hahamy-Dubossarsky A. Dinstein I. Weizman R. Poyurovsky M.. (2011). Global functional connectivity deficits in schizophrenia depend on behavioral state. J. Neurosci. 31, 12972–12981. 10.1523/JNEUROSCI.2987-11.2011 21900576 PMC6623407

[B59] Sanders S. J. He X. Willsey A. J. Ercan-Sencicek A. G. Samocha K. E. Cicek E.. (2015). Insights into autism spectrum disorder genomic architecture and biology from 71 risk loci. Neuron 87, 1215–1233. 10.1016/j.neuron.2015.09.016 26402605 PMC4624267

[B60] Schmahmann J. D. (2010). The role of the cerebellum in cognition and emotion: personal reflections since 1982 on the dysmetria of thought hypothesis and its historical evolution from theory to therapy. Neuropsychol. Rev. 20, 236–260. 10.1007/s11065-010-9142-x 20821056

[B61] Stoddard J. Gotts S. J. Brotman M. A. Lever S. Hsu D. Zarate C.. (2016). Aberrant intrinsic functional connectivity within and between corticostriatal and temporal-parietal networks in adults and youth with bipolar disorder. Psychol. Med. 46, 1509–1522. 10.1017/s0033291716000143 26924633 PMC6996294

[B62] Talairach J. Tournoux P. (1988). Co-planar Stereotaxic Atlas of the Human Brain. New York, NY: Thieme.

[B63] Tan D. W. Gilani S. Z. Mayberry M. T. Mian A. Hunt A. Walters M.. (2017). Hypermasculinised facial morphology in boys and girls with autism spectrum disorder and its association with symptomatology. Sci. Rep. 7:9348. 10.1038/s41598-017-09939-y 28839245 PMC5570931

[B64] Taniai H. Nishiyama T. Miyachi T. Imaeda M. Sumi S. (2008). Genetic influences on the broad spectrum of autism: study of proband-ascertained twins. Am. J. Med. Genet. B Neuropsychiatr. Genet. 147B, 844–849. 10.1002/ajmg.b.30740 18361421

[B65] Wang S. S. H. Kloth A. D. Badura A. (2014). The cerebellum, sensitive periods, and autism. Neuron 83, 518–532. 10.1016/j.neuron.2014.07.016 25102558 PMC4135479

[B66] White E. I. Wallace G. L. Bascom J. Armour A. C. Register-Brown K. Popal H. S.. (2017). Sex differences in parent-reported executive functioning and adaptive behavior in children and young adults with autism spectrum disorder. Autism Res. 10, 1653–1662. 10.1002/aur.1811 28568910 PMC5721669

[B67] Willsey A. J. Sanders S. J. Li M. Dong S. Tebbenkamp A. T. Muhle R. A.. (2013). Coexpression networks implicate human midfetal deep cortical projection in the pathogenesis of autism. Cell 155, 997–1007. 10.1016/j.cell.2013.10.020 24267886 PMC3995413

