# Supplementary material for: Sex Differences in Resting-State Functional Connectivity of the Cerebellum in Autism Spectrum Disorder
Source: Front Hum Neurosci. 2019 Apr 5;13:104. doi: 10.3389/fnhum.2019.00104 (PMC6460665; doi:10.3389/fnhum.2019.00104)
Supplement: Supplementary file 1 [file Data_Sheet_1.docx]

***Supplementary Material***

**1 Evaluating Noise Bias in Seed Voxel Selection**

When performing a two-stage process for the detection of regions using connectedness and follow-up seed testing, noise bias in the individual participant data can potentially affect the regions detected in the post-hoc seed testing (since the same data were used in seed detection and seed testing). This can be evaluated through leave-one-out (LOO) cross validation, defining seeds from connectedness tests for a given participant that excludes their own data. In the current context, 2x2 linear mixed effects models were performed on the whole-brain connectedness maps, leaving each participant out once. The seed locations for each participant corresponded to the peak coordinates within each of the two previously determined cerebellar clusters when not including that participant's data. In this way, the robustness of the previous results using all of the data to noise in the individual participants could be evaluated.

The results for the right cerebellar cluster showed that the peak coordinate was identical when leaving each participant out of the connectedness tests, thereby indicating that the original results for that seed were not strongly affected by noise bias. In contrast, the results for the left cerebellar cluster were modestly affected when leaving each participant out, matching the original peak coordinates 81.6% of the time. This led to a weakening of the seed test results from the left cerebellar seed, with one resulting region surviving correction for 1 test for a voxelwise threshold of P<.005 (and exhibiting a trend when correcting for 2 cerebellar seed tests). For comparison, when using all of the data, this same region survived correction for 2 tests at a voxelwise threshold of P<.0005. However, when combining the results of the two seed tests with LOO cross validation at P<.0005, little was affected overall, since the one resulting region detected from the left cerebellar seed was also detected from the right cerebellar seed. In total, 96.2% of the same voxels were detected at P<.0005 when controlling for noise bias compared to the original results, and none of the 13 resulting regions were lost.
