# Supplementary material for: Sex Differences in Resting-State Functional Connectivity of the Cerebellum in Autism Spectrum Disorder
Source: Front Hum Neurosci. 2019 Apr 5;13:104. doi: 10.3389/fnhum.2019.00104 (PMC6460665; doi:10.3389/fnhum.2019.00104)
Supplement: Supplementary file 2 [file Data_Sheet_2.docx]

**Supplementary Figure 1. Age Distribution by Experimental Group**


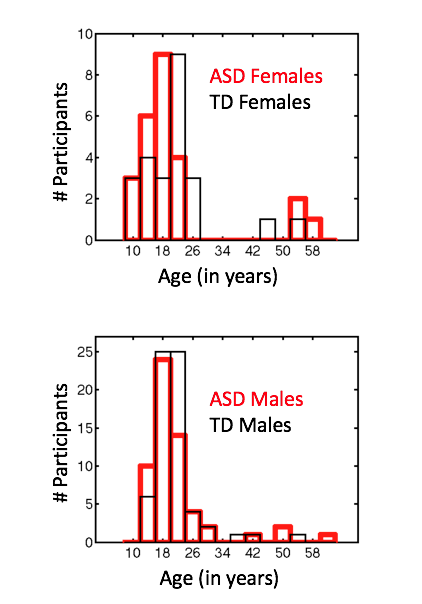


**Supplementary Figure 1:** Histograms of age distribution by group.

X-axis shows age in years, Y-axis shows number of participants

Clinical groups shown in red, control groups shown in black.
